# Accompanying injuries in tibial shaft fractures: how often is there an additional violation of the posterior malleolus and which factors are predictive? A retrospective cohort study

**DOI:** 10.1007/s00068-021-01866-y

**Published:** 2022-01-25

**Authors:** Leonard Lisitano, Edgar Mayr, Kim Rau, Andreas Wiedl, Jan Reuter, Stefan Foerch

**Affiliations:** Universitätsklinikum Augsburg, Klinik Für Unfallchirurgie, Orthopädie, Plastische und Handchirurgie, Stenglinstr. 2, 86156 Augsburg, Germany

**Keywords:** Tibial shaft fracture, Tibial shaft spiral fracture, Posterior malleolus fracture, Accompanying injuries, Low-energy torsion trauma

## Abstract

**Introduction:**

An undislocated fracture of the posterior malleolus is a common concomitant injury in tibial shaft spiral fractures. Nevertheless, these accompanying injuries cannot always be reliably assessed using conventional X-rays. Thus, the aim of the study is to evaluate how often a fracture of the posterior malleolus occurs with tibial shaft fractures (AO:42A/B/C and AO:43A) and which factors—identifiable in conventional X-rays—are predictive.

**Methods:**

Retrospective evaluation of X-ray and CT images revealed a total of 103 patients with low-energy tibial shaft fractures without direct joint involvement. Proximal fractures and fractures involving the knee were excluded. Basic data on injury, the trauma mechanism, the path of the fracture, bony avulsions of the posterior syndesmosis and the procedures performed were evaluated.

**Results:**

Thirty-nine fractures were located in the middle third of the tibia, 64 in the distal third. In 65 cases, a spiral fracture (simple or wedge fracture) was found. In 31/103 fractures, an additional osseous avulsion of the posterior syndesmosis could be detected, 5 (16.1%) of them were not recognized preoperatively due to an absence of CT imaging. In three of these patients, a fracture of the posterior malleolus was only recognized postoperatively, and an additional surgery was necessary. The spiral fractures were classified in the a.p. X-ray according to their path from lateral proximal to medial distal (Type A) or from medial proximal to lateral distal (Type B).

A Pearson chi-square test and Fisher’s exact test showed a highly significant accumulation of accompanying posterior malleolus fractures for type A fractures (*p *= 0.001), regardless of the location of the fracture. In addition, the fractures with involvement of the posterior malleolus had a significantly higher proportion in the fractures of the distal third (*p *= 0.003).

There was no statistically significant relationship between the height of the fracture and the path of the fracture (type A or B). These two factors seem to be independent factors for participation of the posterior malleolus.

**Conclusion:**

In 40.6% of the tibial shaft fractures in the distal third, in 56.9% of the type A spiral fractures and in 67.6% of the type A fractures in the distal third, the ankle joint is involved with bony avulsion of the posterior syndesmosis, which is not always recognized in conventional X-rays. To avoid complications such as additional operations, instability and post-traumatic arthrosis, we recommend preoperative imaging of the ankle using CT for these fractures.

**Level of evidence:**

III, retrospective cohort study.

**Trail registration number:**

DRKS00024536.

## Introduction

Tibial shaft fractures are among the most serious injuries to the lower extremity. In standard diagnostics, X-ray imaging of the lower leg and the adjacent joints (knee and ankle) is performed. This is usually followed by an operative treatment. In many cases, preoperative three-dimensional CT imaging is not carried out, although accompanying ankle joint fractures of the posterior malleolus cannot always be shown with certainty in conventional X-rays, especially in undislocated fractures [1].

The incidence of accompanying fractures of the posterior malleolus (postero-lateral edge) is reported from 1 to 48% in the existing literature [1–6]. Previous studies have already shown that fractures of the posterior malleolus, depending on its size, should be treated surgically to prevent instability, talo-tibial subluxation and thus relevant impairment of the clinical outcome [7–9]. Standard diagnostics must therefore ensure reliable preoperative identification of such accompanying injuries.

The aim of the study is to identify fracture patterns in conventional X-ray that increase the likelihood of an accompanying injury of the posterior malleolus and indicate the need for additional preoperative CT diagnostics.

## Methods

A structured analysis was carried out for all patients with tibial fractures treated at a level I trauma center between January 1, 2018 and April 13, 2021. All pathological fractures, fractures near the knee and all fractures with primary joint involvement as well as all fractures caused by a high-energy trauma were excluded. Fractures without primary joint involvement were defined as all fractures of the tibia, in which the tibial fracture is the main injury, and the path of the main fracture is not continuous in a joint (knee or ankle). Open as well as closed fractures were considered. The exclusion criteria were set to avoid a bias due to special circumstances such as reduced bone quality (pathological fractures), complex trauma mechanisms (multiple trauma) or additional injury to the knee joint.

All available imaging including follow-up examinations were analyzed. In addition, all data on operative care were considered. The fractures were—according to their path in X-ray imaging—graded and classified.

An exploratory data analysis was carried out. The average values were calculated with standard deviations and confidence interval. To determine correlations, the chi-square test for independent samples, Fischer’s exact test and odds ratio for certain factors were calculated. A significance level of *p *≤ 0.05 was set for all significance values.

All statistical tests were performed using SPSS 26 (IBM Germany GmbH, Ehningen, Germany).

The study was classified as ethically harmless by the local ethics committee (Project Nr.: 21–0301) and registered with DKRS/WHO (DRKS00024536).

## Patients

A total of 756 tibial fractures were treated during the study period. 42 women and 61 men—meeting the inclusion criteria—were included in the study. The age varied from 18 to 88 years (mean age 47.9 ± 20.0). According to the AO classification, AO 42A/B/C fractures and AO 43A fractures were examined.

## Results

A total of 103 consecutive tibia fractures in the middle and distal third without direct joint involvement could be included, of which 26 were open and 77 were closed fractures. Patients with previous ankle fractures or existing osteosynthesis as well as poly traumatized patients or patients with previously impaired mobility were excluded.

39 fractures were located in the middle third and 64 fractures in the distal third of the tibia. CT imaging was performed in 54 of 103 cases. A total of 31 posterior malleolus fractures were found, of which 26 could be detected preoperatively. Of the 31 posterior malleolus fractures, 30 occurred with spiral fractures and one with a non-spiral fracture. Preoperative CT imaging was performed for 21 of the 26 patients.

Two posterior malleolus fractures were detected intraoperatively and treated with lag screw osteosynthesis; in three patients, the involvement of the posterior malleolus could only be diagnosed postoperatively. In these cases, an additional surgery was necessary. All five primary unrecognized fractures of the posterior malleolus occurred in spiral fractures. Correspondingly, 16.7% (5/30) of the accompanying fractures were not diagnosed in time due to a lack of CT imaging. Including the above-mentioned cases in which a second operation was necessary, 18 of the 31 fractures (58.1%) of the posterior malleolus fractures required surgical fixation (Table 1).

**Table 1:** Surgical treatment and accompanying fractures of the posterior malleolus according to the primary assessed AO classification

	Total	AO:42A1b	AO:42A1c	AO:42B/C	AO:43A
*n*	103	16	49	23	15
Compression plating	54	5	30	4	15
Intramedullary nailing	49	11	19	19	0
Posterior malleolus fracture (fixation)	31 (18)	5 (3)	25 (14)	0 (0)	1 (1)

65 of 103 fractures were spiral fractures (AO:42A1b/c; AO:43A1.1). Of these, 30 showed an additional posterior malleolus fracture. The Chi-Square test showed a statistically highly significant connection between the fracture morphology (spiral fracture) and an accompanying posterior malleolus fracture (*p *< 0.001).

**Fig. 1:**
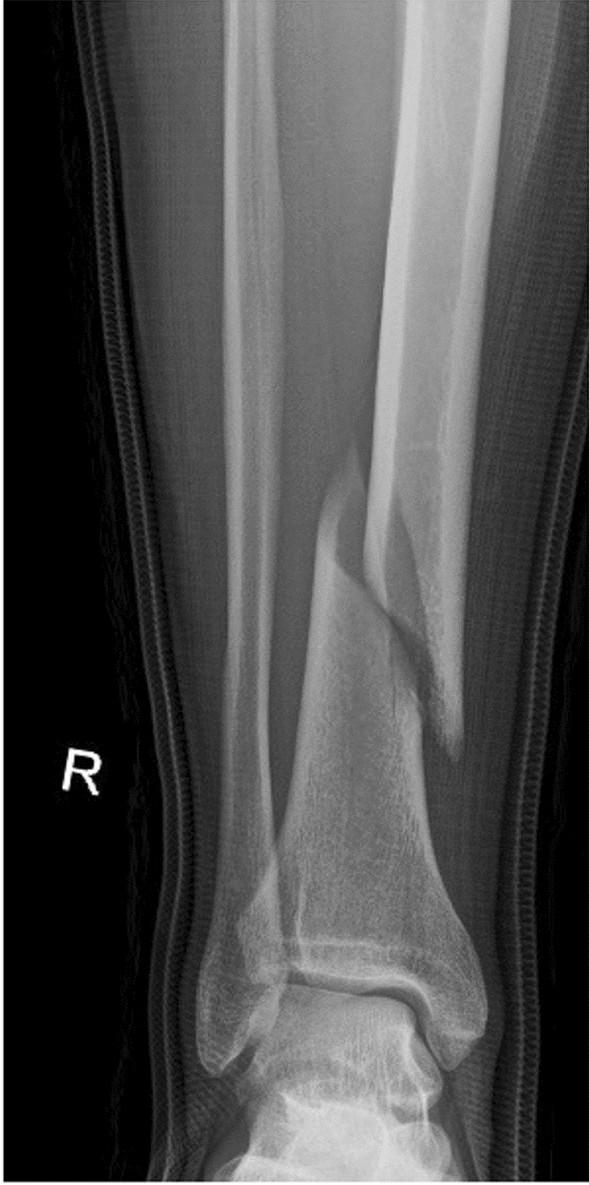
Type A

**Fig. 2:**
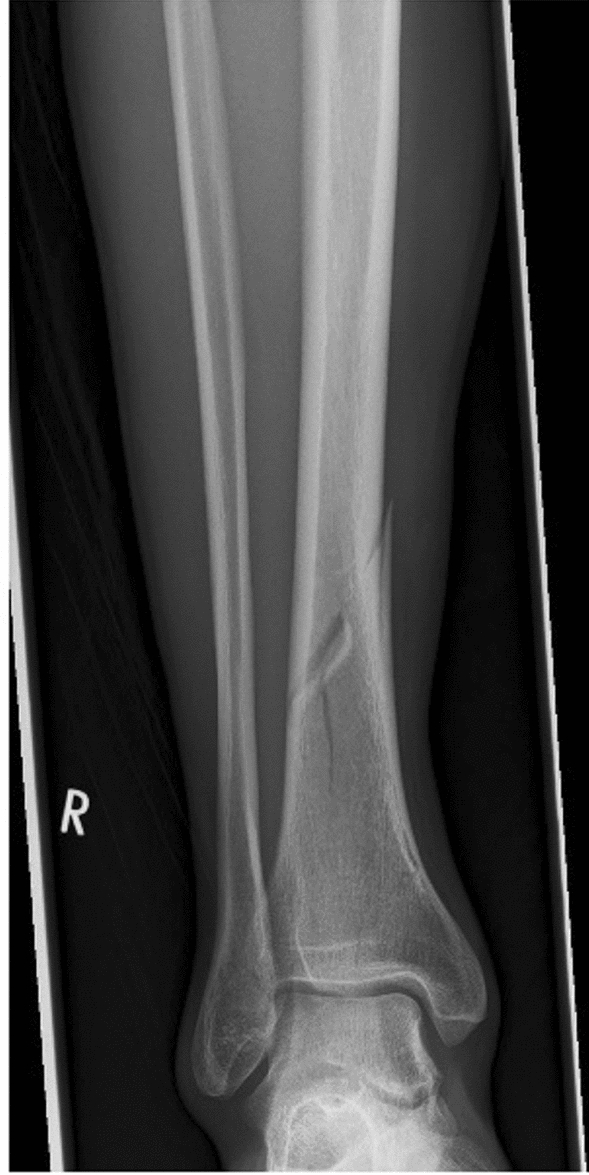
Type B

Spiral fractures were classified according to their path in the a.p. X-ray. A path from proximal lateral to medial distal was designated as “type A”, the reverse path from medial proximal to lateral distal as “type B”. This classification refers only to the AO: 42A1b/c and 43A1.1 fractures and was created by us for better understanding and more fluid readability of the following text (Figs. 1, 2).

For type A fractures (29 of 51), compared to type B fractures (1 of 14), there was a significant increase in the probability of an additional posterior malleolus fracture (*p *= 0.001). This relationship existed for spiral fractures regardless of the fracture location (middle third vs. distal third for spiral type tibia fractures: Chi-square test *p* = 0.168, Fisher’s exact test *p *= 0.249).

In addition, a significant correlation between the fracture localization (for all tibia fractures) and the involvement of the posterior malleolus could be detected (*p* = 0.003). Accompanying injury of the posterior malleolus is more likely in distal third fractures (middle third: 5 of 39 fractures; distal third: 26 of 64 fractures).

However, there was no significant correlation between the fracture location and the path of the fracture (Tables 2, 3).

**Table 2:** Odds ratios and *p* values for various risk factors

Risk factor	Odds ratio	*p* in Chi-square test	*p* in Fisher’s exact test
Spiral fracture	17,813	< 0.001	< 0.001
Fracture in distal third	3,169	0.003	0.004
Type A in spiral fractures	7,961	0.001	0.001

**Table 3:** Number of posterior malleolus fractures per fracture class, as well as the number of cases not recognized preoperatively or only postoperatively

	Total	Spiral fracture	Type A	Type B	Middle third	Distal third
*n*	103	65	51	14	39	64
Posterior malleolus fracture	31	30	29	1	5	26
Preoperative unrecognized	5	5	5	0	1	4
Second operation	3	3	3	0	0	3

The odds ratio was calculated for the risk factors “spiral fracture”, “fracture in the distal third” and “type A in spiral fractures”.

## Discussion

Our findings suggest that fractures with an additional, indirect fracture of the posterior malleolus are mostly uniform injuries with a spiral fracture of the tibia and an undislocated fracture of the posterior malleolus.

Based on the classification of ankle joint fractures by Lauge and Hansen and other biomechanical analyses, the injury is most likely caused by an internal rotation of the ankle against the knee joint (low-energy torsion trauma). An exemplary trauma mechanism would be the rotation of the body outwards with the foot well grounded. This causes the foot to twist inwards, which leads to a spiral fracture of the tibia and, via the traction of the posterior syndesmosis, to a bony tear in the posterior edge of the tibia. However, further investigation and biomechanical testing is required for reliable scientific evidence of this theory.

**Fig. 3:**
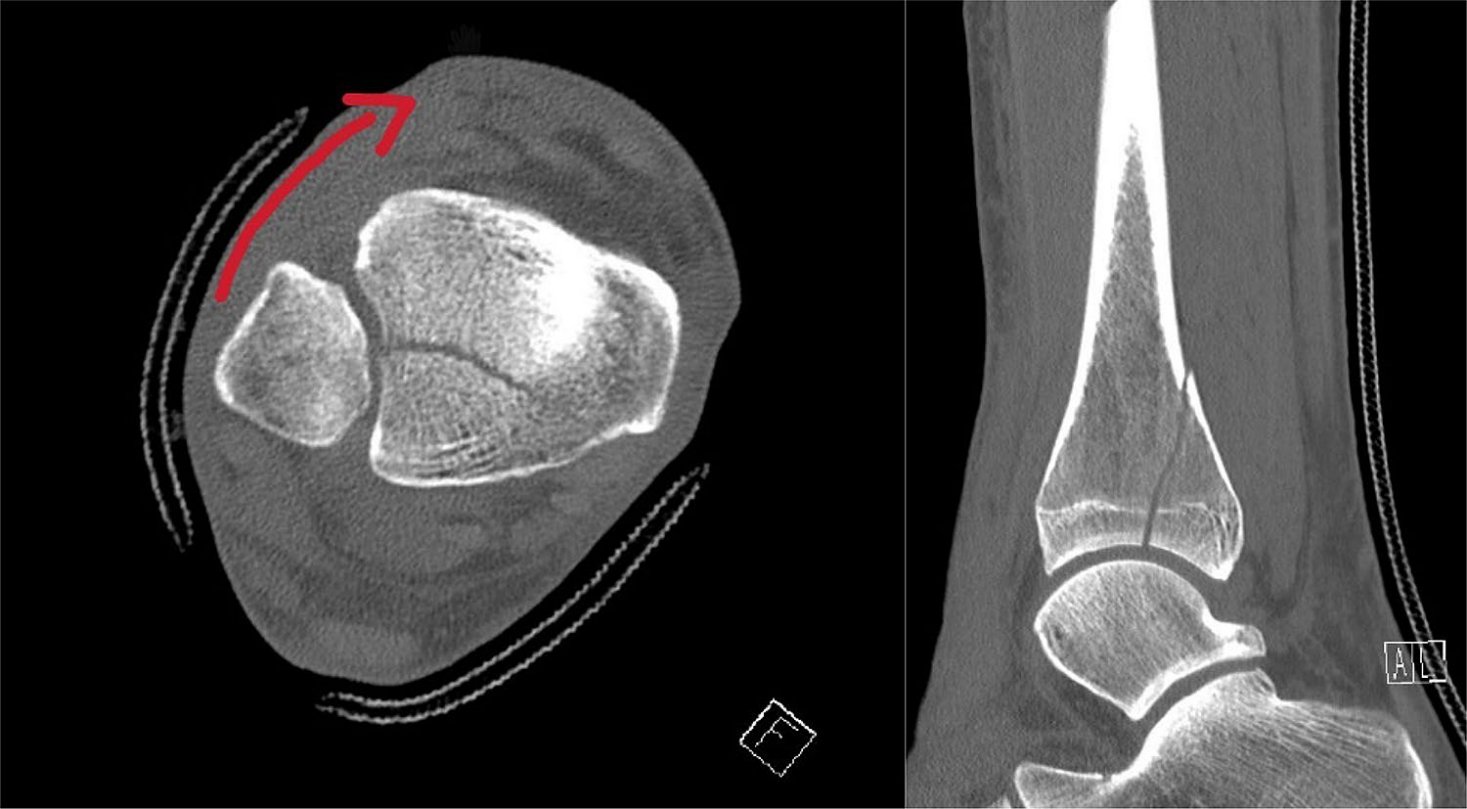
Internal rotation of the ankle causes traction on the posterior malleolus via the posterior syndesmosis

Injury to the posterior malleolus leads to instability in the distal tibio-fibular joint and requires stabilization [7, 10]. Surgical fixation of the posterior malleolus restores stability in the distal tibio-fibular joint. To avoid a dislocation, an intraoperative stability test was only performed after the osteosynthesis. In cases without fixation of the posterior malleolus fractures, an intraoperative stability test was carried out and, if necessary, a positioning screw was inserted (Fig. 3).

In general, the results suggest that most of the existing literature underestimates the incidence of posterior malleolus fractures in tibial shaft fractures [3–5]. Our findings are congruent with a study published by Purnell et al. [1]. Furthermore, even higher rates of occult ankle fractures were found in another study by Warner et al. which performed additional MRI imaging in the absence of accompanying ankle joint involvement [11].

As a non-negligible percentage of the accompanying ankle fractures that require surgical treatment was not recognized in time, it must be concluded that there is a relevant and general issue in standard diagnostics. Several studies have already shown that accompanying fractures of the posterior malleolus cannot be assessed with sufficient certainty using conventional X-rays [12]. In a study by Kukkonen et al., 8/18 concomitant fractures of the posterior malleolus in non-contagious tibial shaft fractures were not recognized preoperatively [4]. In a study by Werken and Zeegers, 8/17 of such accompanying injuries were not diagnosed preoperatively [5]. In a prospective study—examining fractures of the distal third of the tibia without direct joint contact—Purnell et al. discovered that even experienced radiologists (45%, 13/29 not recognized) and orthopedic surgeons (55%, 16/29 not recognized) do not recognize a high proportion of accompanying fractures of the ankle joint in plain X-rays compared to CT imaging. In addition, the same study showed that the size of the posterior tibial edge fragment is mostly underestimated in plain X-rays [1].

Two other recently published studies by Chen et al. and Mitchell et al. showed similar results for spiral fractures in the distal third of the tibia [6, 13]. Spiral tibial shaft fractures, particularly in the middle third of the tibia, were not investigated in these studies.

The results of the present study show that these already known correlations apply to all spiral fractures of the tibia and are not limited to fractures of the distal third.

Various studies on ankle fractures consistently report that diagnosis with conventional X-rays is not sufficient to obtain the necessary surgical information for the posterior malleolar fragment [14, 15]. Supplementary CT imaging to assess the posterior edge issue is recommended in these studies. These results can also be applied to the fractures of the posterior malleolus investigated in this study.

In conclusion, the results of our study and the existing literature demonstrate that both the fracture localization and its path are independent predictive factors for an accompanying injury to the posterior malleolus. In the existing literature, and especially in the present study, there is a highly significant relationship between spiral fractures and fractures of the posterior malleolus. However, none of the previous studies investigated the path of the fracture in spiral fractures. Our investigation suggests that in most cases, it is a uniform injury caused by a low-energy torsion trauma. This leads to a typical fracture path in a.p. X-ray from lateral proximal to medial distal (type A) which predicts an additional injury to the posterior malleolus.

This is clinically relevant considering that Jaskulka et al. demonstrated a significantly poorer long-term outcome for ankle fractures even with small fractures of the posterior tibial edge [8, 16]. Furthermore, in this study, fractures of the posterior malleolus resulted in more frequent and earlier post-traumatic arthrosis of the ankle joint. In addition, various pathoanatomical and biomechanical studies have shown increased instability of ankle fractures with the involvement of the posterior malleolus [17–19]. Since the surgical treatment of posterior malleolus fractures is based on the preoperative diagnosis, a reliable preoperative diagnosis is of the utmost importance for the patient’s outcome.

## Conclusion

An accompanying fracture of the posterior malleolus occurs especially in conjunction with tibial shaft spiral fractures with a path in the a.p. X-ray from lateral proximal to medial distal (type A) as well as in fractures in the distal third of the tibia. On one hand, these additional joint involving fractures cannot always be reliably identified in conventional X-rays. On the other hand, their size is often underestimated. However, surgical treatment of these accompanying fractures is essential for the long-term outcome. We, therefore, recommend supplementary CT imaging with sufficient representation of the ankle joint for all tibial spiral shaft fractures type A and all fractures in the distal third of the tibia.

## Data Availability

The datasets used and analysed during the current study are available from the corresponding author on reasonable request.
